# A LysR Family Transcriptional Regulator Modulates Burkholderia cenocepacia Biofilm Formation and Protease Production

**DOI:** 10.1128/AEM.00202-21

**Published:** 2021-05-26

**Authors:** Kai Wang, Xia Li, Chunxi Yang, Shihao Song, Chaoyu Cui, Xiaofan Zhou, Yinyue Deng

**Affiliations:** a College of Agriculture, South China Agricultural University, Guangzhou, China; b School of Pharmaceutical Sciences (Shenzhen), Sun Yat-sen University, Shenzhen, China; c Institute of Clinical Medicine, Jiangxi Provincial People's Hospital, Nanchang, China; d College of Bioscience and Bioengineering, Jiangxi Agricultural University, Nanchang, China; University of California, Davis

**Keywords:** *Burkholderia cenocepacia*, quorum sensing, LysR family transcriptional regulator, biofilm, protease

## Abstract

Quorum-sensing (QS) signals are widely employed by bacteria to regulate biological functions in response to cell densities. Previous studies showed that Burkholderia cenocepacia mostly utilizes two types of QS systems, including the *N*-acylhomoserine lactone (AHL) and *cis*-2-dodecenoic acid (BDSF) systems, to regulate biological functions. We demonstrated here that a LysR family transcriptional regulator, Bcal3178, controls the QS-regulated phenotypes, including biofilm formation and protease production, in B. cenocepacia H111. Expression of *Bcal3178* at the transcriptional level was obviously downregulated in both the AHL-deficient and BDSF-deficient mutant strains compared to the wild-type H111 strain. It was further identified that Bcal3178 regulated target gene expression by directly binding to the promoter DNA regions. We also revealed that Bcal3178 was directly controlled by the AHL system regulator CepR. These results show that Bcal3178 is a new downstream component of the QS signaling network that modulates a subset of genes and functions coregulated by the AHL and BDSF QS systems in B. cenocepacia.

**IMPORTANCE** Burkholderia cenocepacia is an important opportunistic pathogen in humans that utilizes the BDSF and AHL quorum-sensing (QS) systems to regulate biological functions and virulence. We demonstrated here that a new downstream regulator, Bcal3178 of the QS signaling network, controls biofilm formation and protease production. Bcal3178 is a LysR family transcriptional regulator modulated by both the BDSF and AHL QS systems. Furthermore, Bcal3178 controls many target genes, which are regulated by the QS systems in B. cenocepacia. Collectively, our findings depict a novel molecular mechanism with which QS systems regulate some target gene expression and biological functions by modulating the expression level of a LysR family transcriptional regulator in B. cenocepacia.

## INTRODUCTION

Quorum sensing (QS) is a cell-cell communication mechanism used by various bacteria (1–3). The first, most characterized QS system is the AHL-type system, which usually consists of two components, LuxI and LuxR proteins. The LuxI protein is an autoinducer synthetase that synthesizes the chemical signaling molecule *N*-acyl homoserine lactone (AHL). The LuxR protein is a cytoplasmic autoinducer receptor that contains a DNA-binding transcriptional activation domain. Autoinducers (AHLs) diffuse into the extracellular matrix and accumulate with the increasing number of cells. When the density of the signal reaches a threshold, the signal molecule will bind to the LuxR protein, and then the activated LuxR protein stimulates the expression of target genes (4–7). In addition to AHL-type signals, there are many other kinds of QS signals. One of them is the diffusible signal factor (DSF)-type signal, which was first identified in Xanthomonas campestris (8, 9).

Burkholderia cenocepacia is a major opportunistic pathogen that causes infection in cystic fibrosis and immunocompromised patients (10, 11). It can produce biofilm and numerous virulence factors, including lipopolysaccharide, exopolysaccharide, protease, and toxin. B. cenocepacia possesses the CepIR QS system, which is a LuxIR-type QS system (12). CepI synthesizes two different AHL signals; one is *N*-octanoyl homoserine lactone (C_8_-HSL, OHL), and the other one is *N*-hexanoyl homoserine lactone (C_6_-HSL, HHL). CepR is a homolog of the LuxR protein; it contains two domains, the signal binding domain and the transcriptional activation domain, and uses the same regulatory mechanism as other LuxR-type regulators to control target gene expression (12–16). Furthermore, a fatty acid signal molecule synthesized by B. cenocepacia was identified as *cis*-2-dodecenoic acid, which was also called *Burkholderia* diffusible signal factor (BDSF) (17). Previous study showed that BDSF signal is biosynthesized by B. cenocepacia RpfF (RpfF_BC_). It accumulates in a cell density-dependent manner and regulates the production of various virulence factors. With the bacterial cells gradually accumulating to a high density, BDSF signals bind to RpfR to enhance the phosphodiesterase activity of RpfR and decrease the intracellular cyclic diguanosine monophosphate (c-di-GMP) level, and then they increase the ability of the RpfR-GtrR complex to bind to the promoter DNA of target genes (18). The AHL- and BDSF-type QS systems coordinate to control virulence and physiological functions in B. cenocepacia. In addition, BDSF was also revealed to positively regulate AHL signal production (19).

LysR-type transcriptional regulators (LTTRs) are the most widespread transcriptional regulators in prokaryotes (20). Structures of this type of regulator are conserved and usually contain an N-terminal DNA-binding helix-turn-helix motif and a C-terminal coinducer-binding domain. The coinducing agents are mostly metabolic intermediate substances (21, 22). Increasing evidence suggests that LTTR ShvR controls QS, protease, type II secretion, and colony morphology in B. cenocepacia (23, 24). In this study, we demonstrated that a novel LysR family transcriptional regulator, Bcal3178, was positively controlled by both the AHL and BDSF systems. We also uncovered the regulatory mechanism of Bcal3178 to modulate the phenotypes and QS-regulated target genes in B. cenocepacia H111. In general, our results identify a novel downstream component of the QS systems that help us to further understand the QS signaling hierarchy in B. cenocepacia.

## RESULTS

### Bcal3178 controls QS-regulated phenotypes in B. cenocepacia.

**FIG 1:**
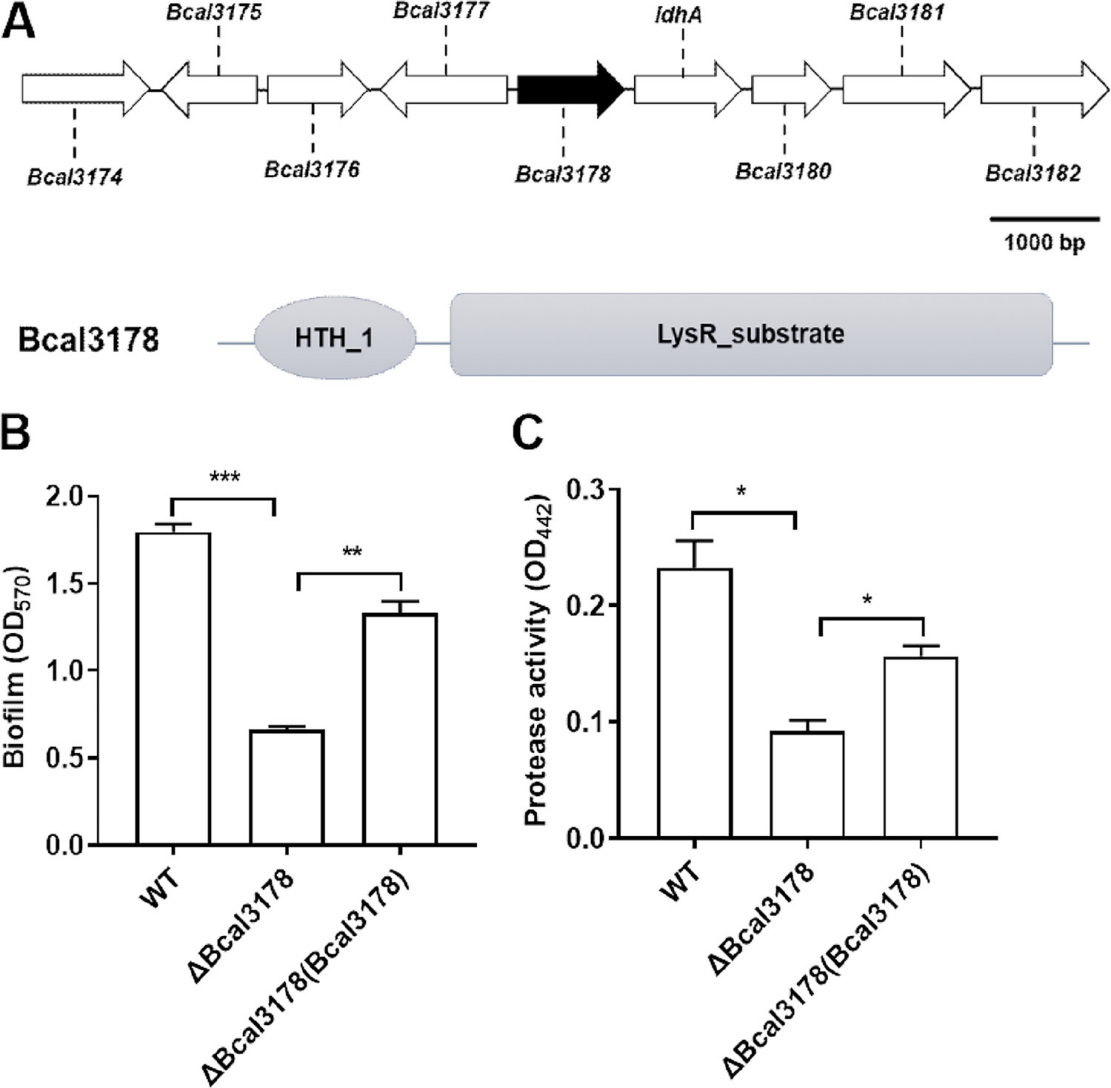
Effects of Bcal3178 on the QS-regulated phenotypes. (A) Genomic organization and domain structure analysis of Bcal3178 in B. cenocepacia H111 (domain structure was analyzed by using the SMART program at http://smart.embl-heidelberg.de). (B and C) Effects of Bcal3178 on biofilm formation (B) and protease activity (C). The data are means ± standard deviations from three independent experiments. *, *P* < 0.05; **, *P < *0.01; ***, *P < *0.001 (unpaired *t* test).

To further investigate the regulatory mechanism, especially the downstream signaling pathways, of the BDSF QS system, we constructed a library of Tn*5* random insertion mutants. The *bclACB* operon is simultaneously regulated by the BDSF and CepIR QS systems in B. cenocepacia H111 (25, 26). Taking advantage of this feature, the *bclACB* operon promoter *lacZ* was fused to a plasmid and transferred into the wild-type B. cenocepacia H111 strain. We screened and identified about 40,000 colonies of the mutant library of B. cenocepacia H111, and the light blue colonies grown in LB agar medium (5 g yeast extract, 10 g tryptone, 10 g NaCl, and 15 g agar per liter) supplemented with X-Gal (5-bromo-4-chloro-3-indolyl β-d-galactopyranoside) were picked out for the identification of insertion sites. Among those identified genes (see Table S1 in the supplemental material), there was a LysR family transcriptional regulator (Bcal3178; I35_RS03450) whose homologues are widely present in many bacteria and play an important role in various physiological activities (27, 28). However, the functions and regulatory mechanisms of Bcal3178 are unclear in B. cenocepacia. Domain structure analysis of Bcal3178 by using the SMART program (http://smart.embl-heidelberg.de/) shows that it has an N-terminal DNA-binding helix-turn-helix motif and a C-terminal coinducer-binding domain (Fig. 1A). In-frame deletion of *Bcal3178* caused a significant downregulation of biofilm formation and protease activity, which are the phenotypes controlled by the two different types of QS systems in B. cenocepacia, and the complemented strain exhibited restored phenotypes (Fig. 1B and C).

### Bcal3178 regulates the target genes by directly binding to the promoter.

**FIG 2:**
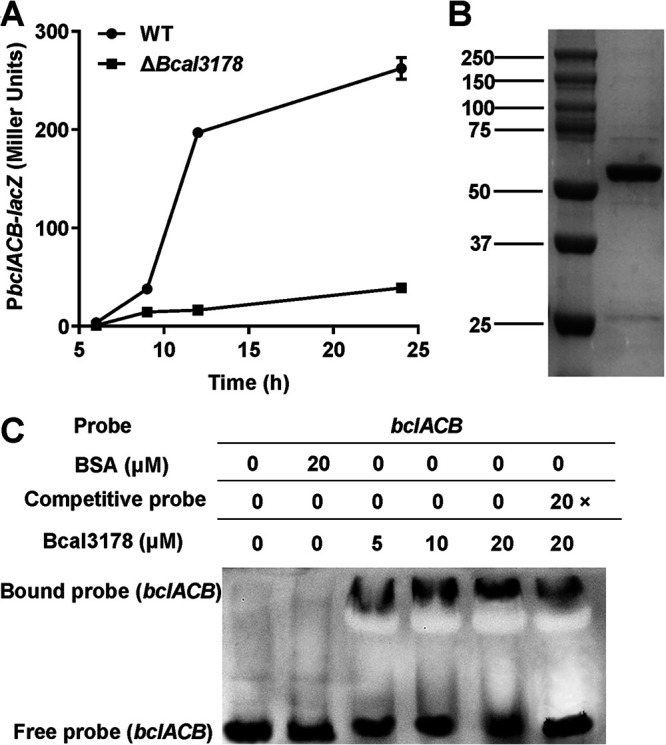
Effects of Bcal3178 on a QS-controlled target gene. (A) The effects of Bcal3178 on the expression level of *bclACB* were measured by assessing β-galactosidase activity of the *bclACB*-*lacZ* transcriptional fusion in the B. cenocepacia H111 wild-type strain (●) and *Bcal3178* deletion mutant strain (■). (B) SDS-PAGE of the GST-Bcal3178 protein. (C) EMSA detection of Bcal3178 binding to the promoter DNA of *bclACB*.

As an insertion mutation of *Bcal3178* resulted in a light blue colony with a *bclACB* operon promoter-*lacZ* fusion plasmid, we supposed that Bcal3178 controls the expression level of the *bclACB* operon. To confirm this speculation, we constructed the P*bclACB-lacZ* reporter system in the *Bcal3178* deletion mutant strain. The expression of *bclACB* in the *Bcal3178* deletion mutant was remarkably lower than that in the wild-type B. cenocepacia H111 strain, as determined by measuring the β-galactosidase activity, suggesting that Bcal3178 positively regulates the expression of *bclACB* (Fig. 2A). To further study whether transcriptional regulation of *bclACB* was achieved by direct binding of Bcal3178 to the promoter, we continued to perform electrophoretic mobility shift analyses (EMSAs) to identify the regulatory mechanism of Bcal3178. A 506-bp DNA fragment of the *bclACB* promoter was obtained by PCR amplification for use as the probe. Bcal3178 is composed of 327 amino acids and was purified using affinity chromatography (Fig. 2B). The EMSAs showed that the complex of Bcal3178 and *bclACB* probe migrated slower than the unbound probe, and the *bclACB* promoter probe that bound to Bcal3178 significantly increased with increasing amounts of Bcal3178 protein (Fig. 2C). Moreover, the amount of labeled probe bound to Bcal3178 decreased in the presence of unlabeled probe (Fig. 2C).

### Transcriptional expression of *Bcal3178* is positively regulated by both the BDSF and AHL QS systems.

**FIG 3:**
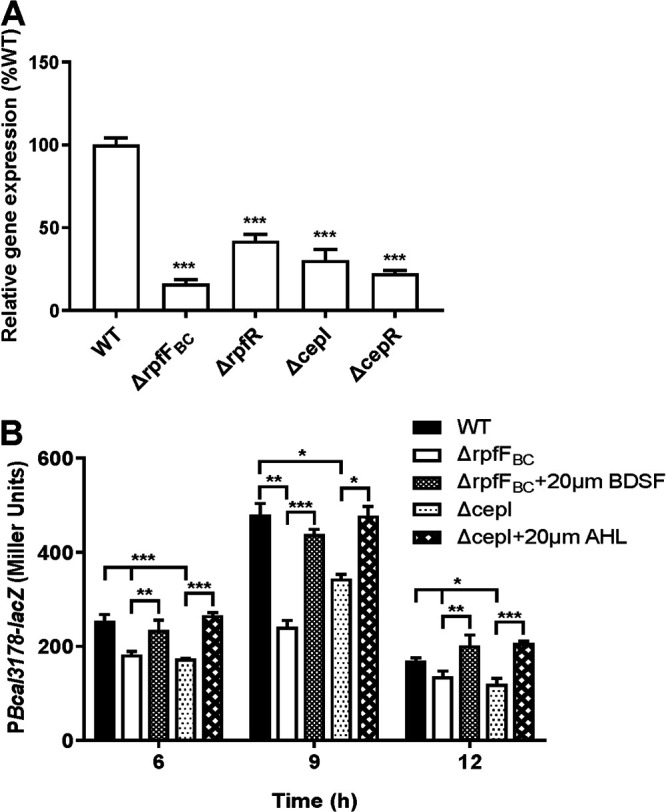
Effects of QS systems on the expression of *Bcal3178*. (A) The expression levels of *Bcal3178* in the wild-type strain and Δ*rpfF_BC_*, Δ*rpfR*, Δ*cepI*, and Δ*cepR* mutant strains were analyzed by using qRT-PCR. The expression level of *Bcal3178* in the wild-type strain was arbitrarily defined as 100% and used to normalize the expression ratios of *Bcal3178* in the mutant strains. (B) The expression levels of Bcal3178 in the wild-type strain and QS signal-deficient mutant strains were measured by assessing β-galactosidase activity of the *Bcal3178*-*lacZ* transcriptional fusions. BDSF and AHL (OHL) were added at a final concentration of 20 μM. The data are means ± standard deviations from three independent experiments. *, *P* < 0.05; **, *P < *0.01; ***, *P < *0.001 (unpaired *t* test).

As *bclACB* operon expression, biofilm formation, and protease activity are regulated by both the BDSF and AHL systems in B. cenocepacia H111, we continued to investigate the relationship between Bcal3178 and the QS systems. We first measured the expression levels of *Bcal3178* in the wild-type H111, BDSF-deficient mutant (*rpfF_BC_* deletion mutant), *rpfR* deletion mutant, AHL-deficient mutant (*cepI* deletion mutant), and *cepR* deletion mutant strains by using quantitative reverse transcription-PCR (RT-PCR) analysis. The results showed that the expression levels of *Bcal3178* of the mutant strains were lower than that of the wild-type H111 strain (Fig. 3A). We then constructed the *Bcal3178-lacZ* reporter system in the wild-type H111, *rpfF_BC_* mutant, and *cepI* mutant strains. The β-galactosidase activity assays revealed that *Bcal3178* expression levels were downregulated in both *rpfF_BC_* and *cepI* mutant strains, and the expression levels were restored with addition of 20 μM BDSF and AHL (OHL), respectively (Fig. 3B).

### Bcal3178 is a downstream component of the BDSF and AHL QS systems.

**FIG 4:**
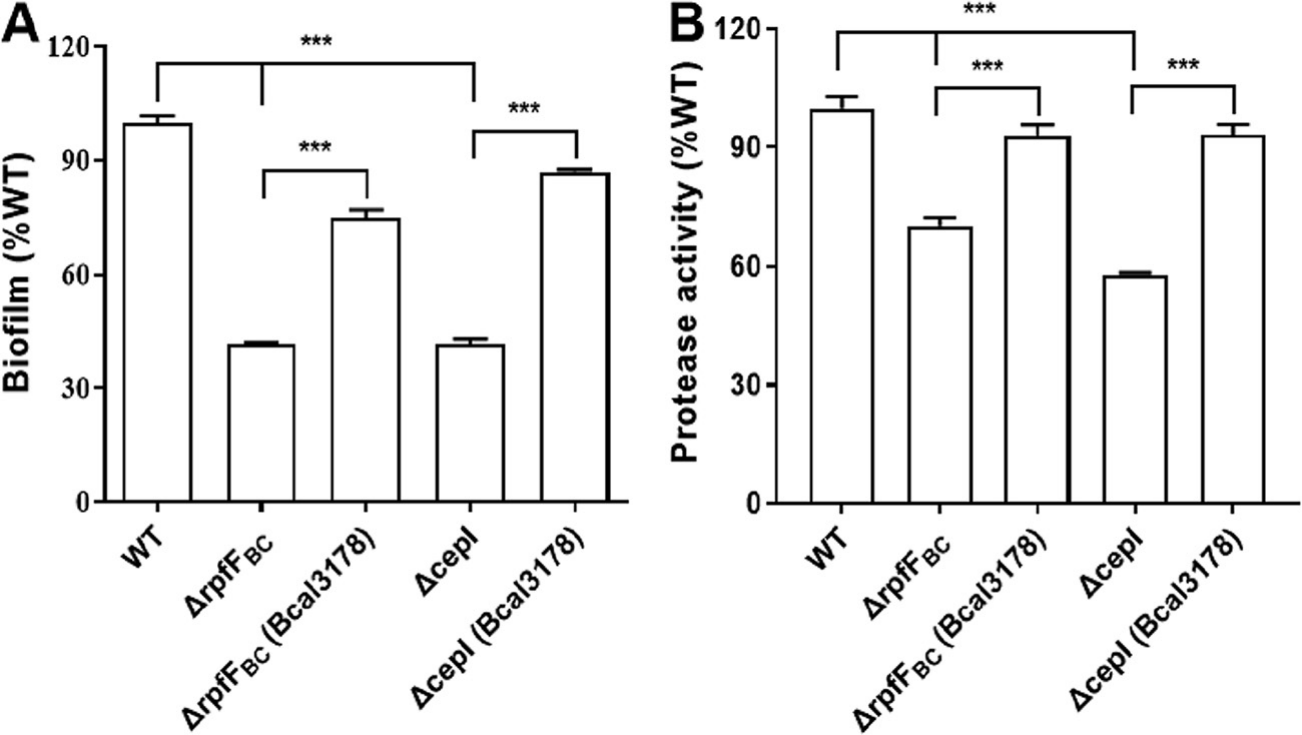
Complementation of the QS signal-deficient mutants with *Bcal3178*. In *trans* expression of Bcal3178 complemented biofilm formation (A) and protease production (B) in the BDSF-deficient Δ*rpfF_BC_* mutant and AHL-deficient Δ*cepI* mutant. The data are means ± standard deviations from three independent experiments. ***, *P < *0.001 (unpaired *t* test).

Based on the facts that Bcal3178 controlled QS-regulated phenotypes and its expression was modulated by QS systems at the transcriptional level, we then expressed *Bcal3178* in *trans* in the *rpfF_BC_* mutant and *cepI* mutant strains. It was shown that in *trans*, the expression of *Bcal3178* in the two mutant strains increased biofilm formation to 75% and 87% of the wild-type strain level (Fig. 4A), and the in *trans* expression of *Bcal3178* in the *rpfF_BC_* mutant and *cepI* mutant strains can almost fully restore protease production to the wild-type strain level (Fig. 4B). These results suggested that Bcal3178 is a downstream component of the QS signaling network in B. cenocepacia.

### CepR regulates *Bcal3178* expression by directly binding to the promoter.

**FIG 5:**
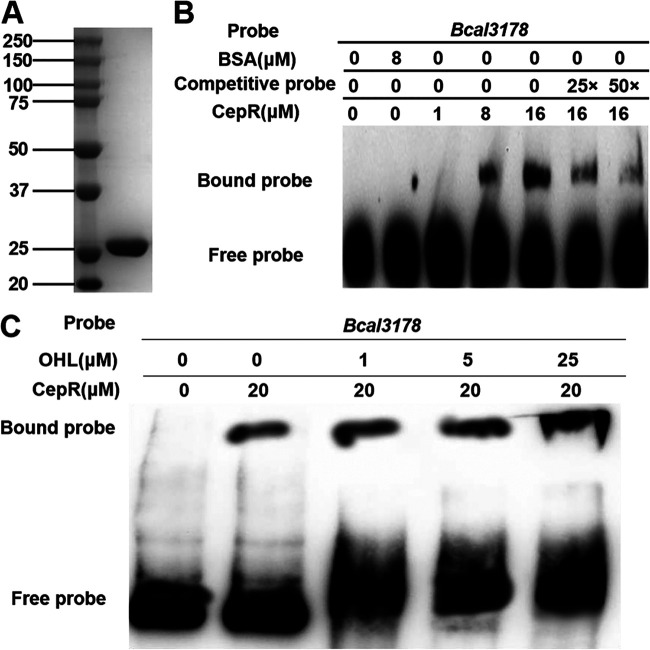
Analysis of the binding between CepR and *Bcal3178* promoters. (A) SDS-PAGE of the CepR protein. (B) EMSA detection of *in vitro* binding of CepR to the promoter of *Bcal3178*, in which a biotin-labeled *Bcal3178* promoter DNA probe was used for protein-binding assays. (C) EMSA detection of *in vitro* binding of CepR to the promoter of *Bcal3178* with the addition of different amounts of AHL (OHL).

To study how the QS systems control *Bcal3178*, we investigated whether transcriptional regulation of *Bcal3178* is achieved by direct binding of regulators of QS systems to the target gene promoter. Since GtrR and CepR are the regulators of the BDSF and AHL systems, respectively, in B. cenocepacia, we used EMSAs to test whether GtrR and CepR can bind the *Bcal3178* promoter. A 223-bp DNA fragment of the *Bcal3178* promoter was obtained by PCR amplification for use as the probe. CepR and GtrR, which are composed of 239 and 463 amino acids, respectively, were purified by affinity chromatography (Fig. 5A and Fig. S1A). It was shown that GtrR did not bind to the probe (Fig. S1B), and the expression levels of *Bcal3178* showed no detectable difference in the wild-type and *gtrR* deletion mutant strains (Fig. S1C and D). CepR, which was purified in the presence of OHL signal, formed a stable DNA-protein complex with the *Bcal3178* promoter DNA fragment, and the migration rate of the complex was slower than that of the unbound probe (Fig. 5B). The amount of labeled probe that bound to CepR increased with increasing amounts of CepR and decreased in the presence of both 25- and 50-fold unlabeled probe (Fig. 5B). Moreover, the binding of CepR to the *Bcal3178* promoter probes was enhanced in the presence of OHL signal (Fig. 5C). These results suggested that CepR is responsible for modulating *Bcal3178* expression. Intriguingly, it was reported that CepR usually binds to the sequence called *lux*-box, which contains the conserved sequence NCTGTNNNGATCNNNCAGNN (12, 15, 27). However, we analyzed the promoter sequence of *Bcal3178* and found no *lux*-box but only a similar sequence, TTCGATACGAGAGCGAAC, in the promoter of *Bcal3178*. Deletion of this fragment from the promoter region of *Bcal3178* did not affect the binding of CepR to the promoter of *Bcal3178* (Fig. S2), suggesting that there is a new binding site for CepR in the promoter region of *Bcal3178*.

### Bcal3178 controls a wide range of QS-regulated genes.

**FIG 6:**
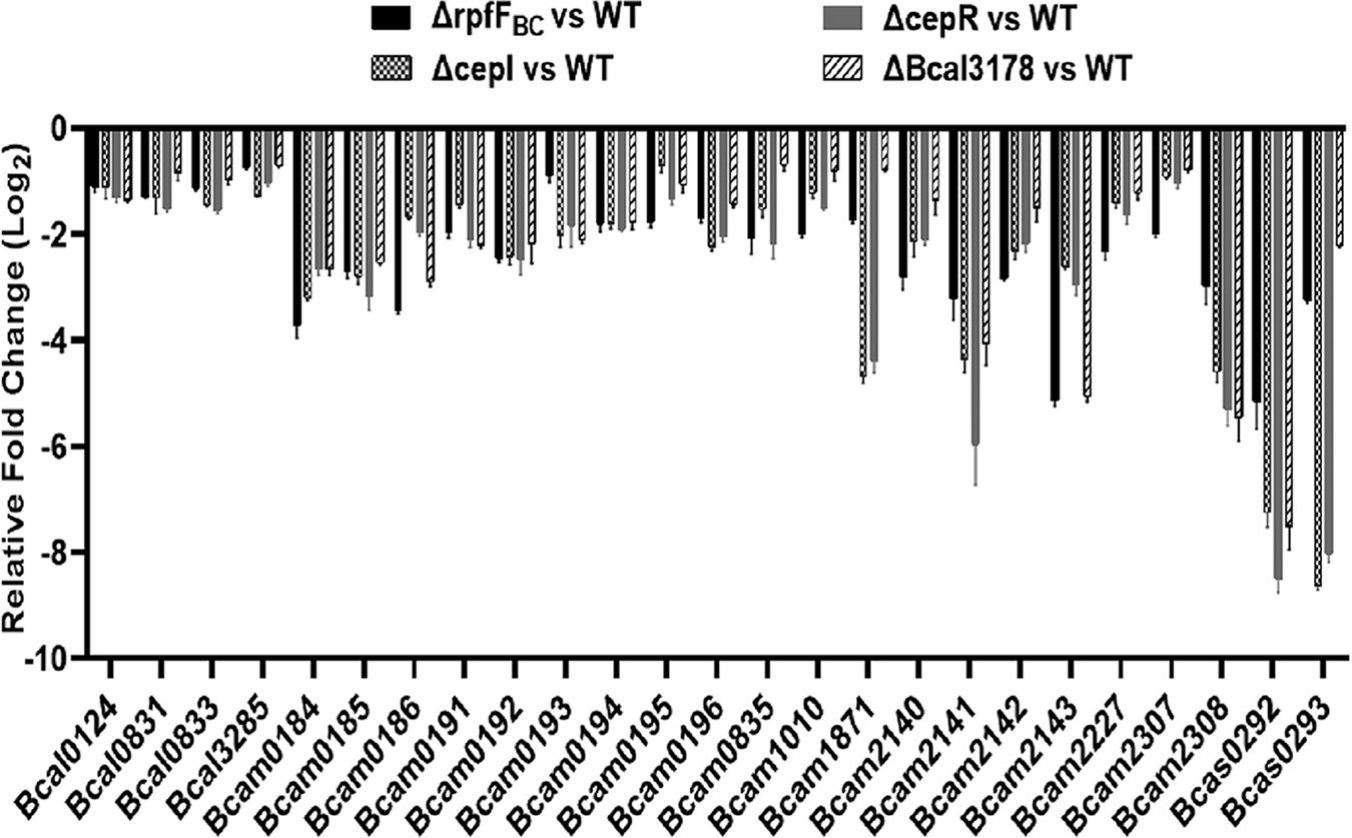
qRT-PCR analysis of the genes that showed differential expression between the Δ*rpfF_BC_*, Δ*cepI*, Δ*cepR*, and Δ*Bcal3178* mutant strains and the wild-type strain. The data are based on three independent experiments, and error bars represent standard deviations.

The BDSF and AHL QS systems control numerous genes and many physiological functions in B. cenocepacia (26). As Bcal3178 is a downstream component of the QS signaling network, we continued to test whether Bcal3178 controls the genes regulated by the BDSF and AHL QS systems (26). Quantitative real-time fluorescence PCR (qRT-PCR) results showed that at least 25 genes were decreased in the *rpfF_BC_*, *cepI*, *cepR*, and *Bcal3178* mutant strains compared with their expression levels in the wild-type H111 strain (Fig. 6). These differentially expressed genes are involved in a range of biological functions (Table S2). However, the expression levels of *rpfF_BC_* and *cepI* showed no detectable differences between the wild-type and *Bcal3178* mutant strains (Fig. S3). These findings suggested that Bcal3178 controls at least a subset of target genes of the QS systems.

## DISCUSSION

In this study, we identified that a LysR family transcriptional regulator, Bcal3178, is a new global transcriptional regulator controlling various gene expression and biological functions (Fig. 1, 2, and 6). Intriguingly, both of these genes and biological functions are coregulated by the BDSF and AHL systems in B. cenocepacia. Moreover, the expression levels of *Bcal3178* were significantly downregulated in the QS-deficient mutant strains compared to the wild-type B. cenocepacia H111 strain (Fig. 3), while Bcal3178 exhibited no detectable effect on the expression levels of BDSF or AHL signal synthase-encoding genes (Fig. S3). Previous studies showed that another LysR family transcriptional regulator, ShvR, was controlled by the AHL QS system in B. cenocepacia K56-2 (23, 24). ShvR also influenced the production of a set of virulence factors and AHL signal production in B. cenocepacia K56-2 (23, 24), suggesting the distinguishing roles of Bcal3178 from other LysR family transcriptional regulators in B. cenocepacia.

**FIG 7:**
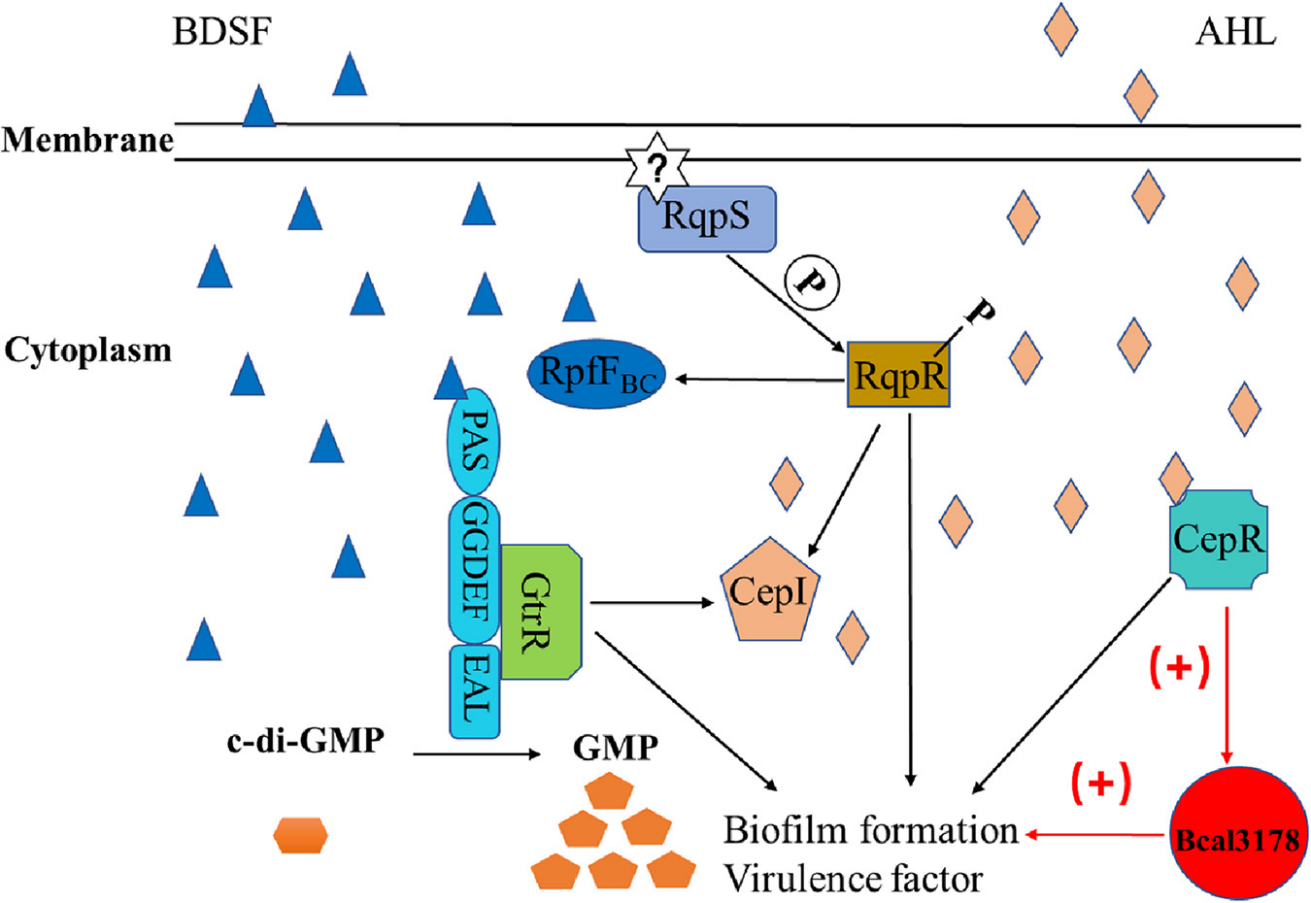
Schematic diagram of the QS signaling network in B. cenocepacia. The two-component system RqpSR positively regulates expression of the *cepI* and *rpfF_BC_* genes, which are required for the synthesis of the BDSF and AHL signals, respectively. Binding of BDSF to the receptor RpfR substantially increases its c-di-GMP degradation activity and results in a reduced intracellular c-di-GMP level and, consequently, affects *cepI* transcriptional expression. CepR, activated by AHL signals, directly binds to the promoter of *Bcal3178* and enhances the expression of Bcal3178, which finally controls some QS-regulated target gene expression and biological functions. Solid arrows indicate regulation or signal transduction.

It was already demonstrated that the AHL and BDSF QS systems are not completely independent and form a signaling network (19). The two QS systems coregulate various genes and phenotypes. BapA (encoded by BCAM2143) is a large surface protein that plays a vital role in biofilm formation (29, 30). A cluster of three genes, *bclACB* (BCAM0184 to -0186), encode lectins, which are also needed for biofilm structural development (26, 29). ZmpB is a zinc metalloprotease that is a vital component of proteolytic activity (31). All of these genes were significantly downregulated in both the BDSF-deficient and AHL-deficient mutant strains, as previously reported (26), and were downregulated in the *Bcal3178* deletion mutant strain compared to the wild-type H111 strain (Fig. 6). In addition, the deletion of *Bcal3178* impaired biofilm formation and protease production, while in *trans* expression of *Bcal3178* restored the biofilm formation and protease production of both the QS signal-deficient mutants and the *Bcal3178* deletion mutant (Fig. 1 and 4). Moreover, the substantially overlapping genes controlled by both the BDSF and AHL systems were regulated by Bcal3178 (Fig. 6). Taken together, these findings support that Bcal3178 is a novel key downstream component of the QS signaling network in B. cenocepacia (Fig. 7).

Several previous reports showed that LTTR was controlled by the AHL-dependent QS system, but the regulatory mechanism is unclear (23, 27). In this study, we discovered that CepR, the receptor of the AHL system, directly bound to the promoter of *Bcal3178*, and OHL signal enhanced the binding activity (Fig. 5B and C). From these results, we can propose a new regulatory mechanism in which the AHL signals accumulate to the threshold and bind to CepR, and then the activated CepR regulates Bcal3178 by directly binding to the promoter of *Bcal3178*, thereby controlling target gene expression as well as biofilm formation and protease phenotypes (Fig. 7). Nevertheless, a new issue is that we did not find an obvious *lux*-box sequence in the promoter region of *Bcal3178*. Furthermore, the BDSF QS system usually regulates target genes through the BDSF-RpfR-GtrR complex (18), but the EMSA result showed that there was no binding between GtrR and the *Bcal3178* promoter (see Fig. S1B in the supplemental material), and the expression levels of *Bcal3178* were similar in the wild-type and *gtrR* deletion mutant strains (Fig. S1C and D), suggesting that BDSF employs another novel regulator or the AHL QS system to regulate *Bcal3178* expression, which needs to be investigated further (Fig. 7).

## MATERIALS AND METHODS

### Bacteria strains and growth conditions.

All the strains used in this study are listed in Table 1. B. cenocepacia H111 strains and Escherichia coli were grown at 37°C in LB medium (5 g yeast extract, 10 g tryptone, and 10 g NaCl per liter; solid medium also contains 15 g agar per liter). In this work, antibiotics were used at the following concentrations: ampicillin, 100 μg/ml; kanamycin, 100 μg/ml; gentamicin, 50 μg/ml; and tetracycline, 20 μg/ml. The chromogenic substrate X-Gal (5-bromo-4-chloro-3-indolyl β-d-galactopyranoside) was used at 40 μg/ml. Bacterial growth was monitored spectrophotometrically by measuring the optical density at a wavelength of 600 nm (OD_600_).

**TABLE 1:** Bacterial strains and plasmids used in this study

Strain or plasmid	Phenotype and/or characteristics^*a*^	Reference or source
B. cenocepacia
H111	Wild-type strain, genomovar III of the B. cepacia complex	34
Δ*rpfF_BC_*	BDSF-minus mutant derived from H111 with *rpfF_BC_* deleted	17
Δ*rpfR*	Deletion mutant with *rpfR* deleted	38
Δ*cepI*	Deletion mutant with *cepI* deleted	19
Δ*cepR*	Deletion mutant with *cepR* deleted	12
Δ*gtrR*	Deletion mutant with *gtrR* deleted	18
Δ*Bcal3178*	Deletion mutant with *Bcal3178* deleted	This study
Δ*rpfF_BC_*(*Bcal3178*)	Δ*rpfF_BC_* mutant harboring expression construct pBBR1-mcs2*-Bcal3178*	This study
Δ*cepI*(*Bcal3178*)	Δ*cepI* mutant harboring expression construct pBBR1-mcs2-*Bcal3178*	This study
Δ*Bcal3178*(*Bcal3178*)	Δ*Bcal3178* mutant harboring expression construct pBBR1-mcs2*-Bcal3178*	This study
H111(P*bclACB*-*lacZ*)	H111 harboring reporter construct P*bclACB*-*lacZ*	18
Δ*Bcal3178*(P*bclACB*-lacZ)	Δ*Bcal3178* mutant harboring reporter construct P*bclACB*-*lacZ*	This study
H111(P*Bcal3178*-*lacZ*)	H111 harboring reporter construct P*Bcal3178*-*lacZ*	This study
Δ*cepI*(P*Bcal3178*-*lacZ*)	Δ*cepI* mutant harboring reporter construct P*Bcal3178*-*lacZ*	This study
Δ*rpfF_BC_*(P*Bcal3178*-*lacZ*)	Δ*rpfF_BC_* mutant harboring reporter construct P*Bcal3178*-*lacZ*	This study
Δ*gtrR*(P*Bcal3178*-*lacZ*)	Δ*gtrR* mutant harboring reporter construct P*Bcal3178*-*lacZ*	This study
E. coli
DH5α	*supE44 lacU169*(80*lacZ*M15) *hsdR17 recA1 endA1 gyrA96 thi-1 relA1 pir*	Laboratory collection
BL21	F^−^ *ompT hsdS* (r_B_^−^m_B_^−^) *dcm*^+^ Tet^r^ *gal*(DE3) *endA*	Stratagene
Plasmids
pBBR1-mcs2	Broad-host-range cloning vector; Kan^r^	Laboratory collection
pBBR1-mcs2*-Bcal3178*	pBBR1-mcs2 containing *Bcal3178*	This study
pK18	pK18, *sacB*^+^; gene replacement vector; Kan^r^	Laboratory collection
pK18-*Bcal3178*	pK18 containing fragments flanking *Bcal3178* and Gm-resistant fragment; Kan^r^, Gm^r^	This study
pRK2013	RK2 derivative, *mob*^+^ *tra*^+^ *ori* ColE1; Kan^r^	39
pME2-*lacZ*	Transcriptional level reporter vector; Tet^r^	Laboratory collection
P*bclACB*-*lacZ*	pME2-*lacZ* containing promoter of *bclACB*	18
P*Bcal3178*-*lacZ*	pME2-*lacZ* containing promoter of *Bcal3178*	This study
pGEX-6p-1	Expression vector; Amp^r^	Amersham
pGEX-*Bcal3178*	pGEX-6p-1 containing *Bcal3178*	This study
pDBHT2	Expression vector; Kan^r^	Laboratory collection
pDBHT2-*cepR*	pDBHT2 containing *cepR*	This study
pDBHT2-*gtrR*	pDBHT2 containing *gtrR*	This study

a Kan^r^, Tet^r^, Amp^r^, and Gm^r^ indicate resistance to kanamycin, tetracycline, ampicillin, and gentamicin, respectively.

### Screening and identification of mutants in which Tn*5* was randomly inserted.

A mini-Tn*5* transposon with a gentamicin resistance gene was transformed into B. cenocepacia H111 with the *bclACB* operon promoter-*lacZ* fusion by triparental mating. The transformants were selected on LB plates supplemented with X-Gal and gentamicin. The light blue colonies were picked out for the identification of insertion sites. High-efficiency thermal PCR was used to identify DNA flanking sequences at the insertion site of the Tn*5* transposon as previously described (32).

### Construction of in-frame deletion mutant and complemented strains.

B. cenocepacia H111 was used as the parental strain to construct the *Bcal3178* deletion mutant by following previously described methods (17). The upstream and downstream fragments of *Bcal3178* were generated by using the two PCR primer pairs listed in Table 2. For the generation of complementation, the coding region of *Bcal3178* was amplified and cloned into the plasmid pBBR1-MCS2. The resulting construct was conjugated into the B. cenocepacia H111 Δ*Bcal3178* deletion mutant using triparental mating with pRK2013 as the mobilizing plasmid. The construct was also conjugated into B. cenocepacia H111 Δ*rpfF_BC_* and Δ*cepI* deletion mutants using the same methods (33).

**TABLE 2:** PCR primers used in this study

Primer	Sequence^*a*^ (5′–3′)
For deletion
* Bcal3178* L-F	CTATGACATGATTACGAATTCCGCTCGTTGATTAGGTGGTGT
* Bcal3178*L-R	TTCCACGGTGTGCGTCCACTGCGCGCGTCAGCCATCGGA
* Bcal3178*R-F	TAAATTGTCACAACGCCGCCGGCTTGCGTATTTCTGGCC
* Bcal3178*R-R	CTGCCGTTCGAATCCCACGGCGCAGCGAACTGA
* Bcal3178*GM-F	AGTGGACGCACACCGTGGAAA
* Bcal3178*GM-R	GGCGGCGTTGTGACAATTT
* Bcal3178*IN-F	GTACTGGCGGTTCGGATAGA
* Bcal3178*IN-R	CACCTGAACACACGGCTGAT
* Bcal3178*OUT-F	TCCGCTCGTTGATTAGGTGG
* Bcal3178*OUT-R	ATGAGGAAAGGAAGTGCCCG
For *in trans* expression and reporter
* Bcal3178*C-F	GGGGTACCATGAACCAGATTCAGACCATGCG
* Bcal3178*C-R	GCTCTAGATTACTGCAGGCCCGTGACG
P*bclACB*-F	CCGCTCGAGCGGAATCTGGCGCTTCAGGAAAGAA
P*bclACB*-R	CCCAAGCTTGGGGCGGTTGGATGACGTTTGAGA
P*Bcal3178*-F	CCCAAGCTTATATTCGAATACCGCGACGG
P*Bcal3178*-R	CCGCTCGAGATTGGACACGCCGAGATGGT
For EMSA
EMSA-*bclACB*-F	GATGTCGGTCCTCGGTCT
EMSA-*bclACB*-R	CGAACATGAATAGGGCCT
EMSA*-Bcal3178*-F	TGCTGCATTGCAACCTTA
EMSA*-Bcal3178*-R	GGCTTGCGTATTTCTGGCC
For recombinant protein
* Bcal3178*-GST-F	CGGGATCCATGAACCAGATTCAGACCATGCG
* Bcal3178*-GST-R	CGGAATTCTTACTGCAGGCCCGTGACG
* cepR*-HIS-F	CGGGATCCATGGAACTGCGCTGGCAG
* cepR*-HIS-R	CGGAATTCTCAGGGTGCTTCGATGAG
* gtrR*-HIS-F	CGGGATCCATGAGAAATACGCCCGCAAT
* gtrR*-HIS-R	CGGAATTCTTACTCGCTTTCGCGGGTCT
For RT-qPCR
* Bcal3178*-F	CATGCGTGTATTCGTCTGCG
* Bcal3178*-R	TGGATCAGCCGTGTGTTCAG
* cepI*-F	AGTTCGATCGCGACGATACC
* cepI*-R	AGCGACTTCAGCAGATACGG
* rpfF_BC_*-F	CACGTTCGACTTCTGGGTGA
* rpfF_BC_*-R	CCGAAGCCCGTGTAGATCTC
* recA*-F	GTACGATCAAGCGCACGAAC
* recA*-R	GATCCGGCGGATATCGAGAC
* BCAL0124*-F	ACCTGTCGTACCTCCTCCTC
* BCAL0124*-R	CGTGATCATCGAAGCGGAAG
* BCAL0831*-F	TCCGTATTTGCCCCCGAAAA
* BCAL0831*-R	TTGCAGGTTGAGTTCGACGA
* BCAL0833*-F	TAGTCGTCACGTATTCGCCG
* BCAL0833*-R	CTTCTCGATGCATTGCTGGC
* BCAL1063*-F	ACAACGACGTGATCTCGGTC
* BCAL1063*-R	TGAACAGGTACGACGTCACC
* BCAL2353*-F	CTGTTCCGCTCGGTGATGAA
* BCAL2353*-R	AGCAGGAAGTGGTCGTCATG
* BCAL3285*-F	ACATCACCGCTGACCAGATG
* BCAL3285*-R	TGCGTCTGGTTGAACAGGTT
* BCAM0184*-F	CAACCCTTTACCCACGACGA
* BCAM0184*-R	CGTATTGCGGCAGTTTCTCG
* BCAM0185*-F	CCCTCCTTTCGGCTTCGATT
* BCAM0185*-R	GCGATCGCGAAATAGATGCC
* BCAM0186*-F	CTCAAACGTCATCCAACCGC
* BCAM0186*-R	GCTGTCGCCGATGAACAATT
* BCAM0191*-F	TGACCGATTCGACGCTTCAA
* BCAM0191*-R	GAAATACTCGGCCGCGTAGA
* BCAM0192*-F	CGTGTGGGATTTCATGTCGC
* BCAM0192*-R	AGGTACAGGTCGTAGTGGCT
* BCAM0193*-F	GCACGACTACCACGAGGAAG
* BCAM0193*-R	GAAGTAGCTGCCTTCCCGAT
* BCAM0194*-F	TTCCTGCGCGAATACCTGAG
* BCAM0194*-R	TGACGATCATCGGATGCTGG
* BCAM0195*-F	ACGTCGTCGCGTTCTATCTC
* BCAM0195*-R	GATAGCCGAAATGCGCATCG
* BCAM0196*-F	GCTCGACCATACCGACATGA
* BCAM0196*-R	CGACGTATGGATCAGGCTCC
* BCAM0835*-F	GTGAACCGCATCTCGATTGC
* BCAM0835*-R	CAGCGTCGTATGGATCAGCA
* BCAM1005*-F	GAACACGCCGATGTCGAATG
* BCAM1005*-R	GTAGACGGTGTAGCTGACGG
* BCAM1010*-F	TGTCGGGCATCATCGAGAAG
* BCAM1010*-R	GCTTGCGCAGATGATCGAAG
* BCAM1745*-F	CCGACATCATCCTGCTCGAA
* BCAM1745*-R	TGGCCGTCATGTTCAGGTAC
* BCAM1871*-F	CTCGAACGACAGGTTGACGA
* BCAM1871*-R	GTATTTGCTGCGCATCTCCG
* BCAM2060*-F	GTGCTGTACGTGAACCAGGA
* BCAM2060*-R	GTTGAGCAGGAACAGGTCGA
* BCAM2140*-F	AATTCTCGACGAAGCTCGCA
* BCAM2140*-R	GATGTCTTTCACGATGCCGC
* BCAM2141*-F	CGATCATTTCGGCAAGCAGG
* BCAM2141*-R	GACGAACGGGATGTCGATCA
* BCAM2142*-F	GAACCGTGAAAGCCTCGAGA
* BCAM2142*-R	GCGGTCACTTTCTCGTAGCT
* BCAM2143*-F	GACGATCCAGGTCGATGGTC
* BCAM2143*-R	GTATCCACCACGATCCCCAC
* BCAM2169*-F	GTACACGTGGTACCGCATCA
* BCAM2169*-R	GTTTCCGTATAGCCGTCGGT
* BCAM2227*-F	ACAGGAAGGCTTGTCGGAAG
* BCAM2227*-R	CGTCCCAGTTGTAGACCCAG
* BCAM2307*-F	GATGGACAAGGCGTTCCTGA
* BCAM2307*-R	GTGCAGCTCTTGTTGTACGC
* BCAM2308*-F	GCCTACTCTGAAACCGACCC
* BCAM2308*-R	CATCGATGCGTTGAAGCTGG
* BCAS0292*-F	GTCTGGTGTTCGTTGCGATG
* BCAS0292*-R	CAAAGAGCCGGTTGTCGTTG
* BCAS0293*-F	ATGTCACGCGTTACCGATGT
* BCAS0293*-R	GACATAGCGCCAGTCGATCA

a Restriction enzyme sites are underlined.

### Biofilm formation and protease activity assays.

The bacterial cells were cultured overnight and diluted to an OD_600_ of 0.01 by using minimal medium [per liter, 2 g glycerin, 2 g mannitol, 10.5 g K_2_HPO_4_, 4.5 g KH_2_PO_4_, 2 g(NH_4_)_2_SO_4_, 0.2 g MgSO_4_·7H_2_O, 0.005 g FeSO_4_, 0.01 g CaCl_2_, 0.002 g MnCl_2_]. Biofilm formation in 96-well polypropylene microtiter dishes was performed as described previously (34). For analyzing the protease activity, bacteria were cultured in NYG medium (per liter, 3 g yeast extract, 5 g peptone, 20 g glycerin) overnight at 37°C with shaking at 200 rpm, diluted to an OD_600_ of 0.01 in NYG, and then cultured at 37°C with shaking at 200 rpm for 18 h. Protease activity was determined by following previously published methods (35).

### Construction of reporter strains and measurement of β-galactosidase assays.

The *bclACB* reporter was introduced into the B. cenocepacia H111 and Δ*Bcal3178* mutant strains by electroporation. The *Bcal3178* reporter was introduced into the B. cenocepacia H111, Δ*rpfF_BC_*, Δ*gtrR*, and Δ*cepI* strains by triparental mating. The transconjugants were selected on LB agar plates supplemented with ampicillin, tetracycline, and X-Gal. For measurement of β-galactosidase activities, the overnight-cultured bacteria were diluted to the same cell densities (OD_600_, 0.01) in LB medium supplemented with ampicillin and tetracycline. The inoculated cultures were then incubated at 37°C with shaking at 200 rpm and harvested to measure β-galactosidase activities by following previously described methods (36).

### Protein expression and purification assays.

The coding regions of *Bcal3178*, *gtrR*, and *cepR* were amplified with the primers listed in Table 2 and ligated to the expression vectors pGEX-6p-1 and pDBHT2, as indicated. The resulting constructs were transformed into E. coli BL21. The bacteria were cultured in LB medium with ampicillin and kanamycin, respectively, and the strain with pDBHT2-cepR was cultured with the addition of OHL (50 nM) (15). Affinity purifications of GST-Bcal3178, HIS-GtrR, and HIS-CepR fusion proteins were performed by following methods described previously (33). The fusion proteins were eluted and verified by SDS-PAGE.

### EMSA.

The DNA probes used for electrophoretic mobility shift assay (EMSA) were harvested by PCR amplification using the primer pairs listed in Table 2. The purified PCR products of *bclACB* and *Bcal3178* promoters were 3′-end labeled with biotin according to the manufacturer’s instructions (Thermo). The biotin-labeled probes and proteins were prepared for the DNA-protein binding reactions by following the manufacturer’s instructions (Thermo). A 5% polyacrylamide gel was used to separate the DNA-protein complexes from the unbound probes by following methods described previously (33). After UV cross-linking, the biotin-labeled probes were detected in the membrane, with different mobilities for the bound probes and unbound probes.

### Quantitative real-time fluorescence PCR.

The bacterial cells were collected by centrifuging at 13,000 rpm for 2 min after growth to an OD_600_ of 1.0. The total RNA was prepared using an RNA extraction kit (Promega). Reverse transcription-PCR was performed using a cDNA synthesis kit (Promega) according to the manufacturer’s instructions. The qRT-PCR assays were performed using a SYBR green qPCR master mix (Thermo Scientific) and a 7300Plus real-time PCR system (Applied Biosystems). *recA* was used as the control. The relative expression levels of different target genes were analyzed by following the quantitation-comparative threshold cycle (ΔΔ*C_T_*) method as described previously (37).
