# Supplementary material for: A LysR Family Transcriptional Regulator Modulates Burkholderia cenocepacia Biofilm Formation and Protease Production
Source: Appl Environ Microbiol. 2021 May 26;87(12):e00202-21. doi: 10.1128/AEM.00202-21 (PMC8174753; doi:10.1128/AEM.00202-21)
Supplement: SUPPLEMENTAL FILE 1 — Supplemental material. Download aem.00185-21-s0001.pdf, PDF file, 1.1 MB [file aem.00185-21-s0001.pdf]

Supplemental Material

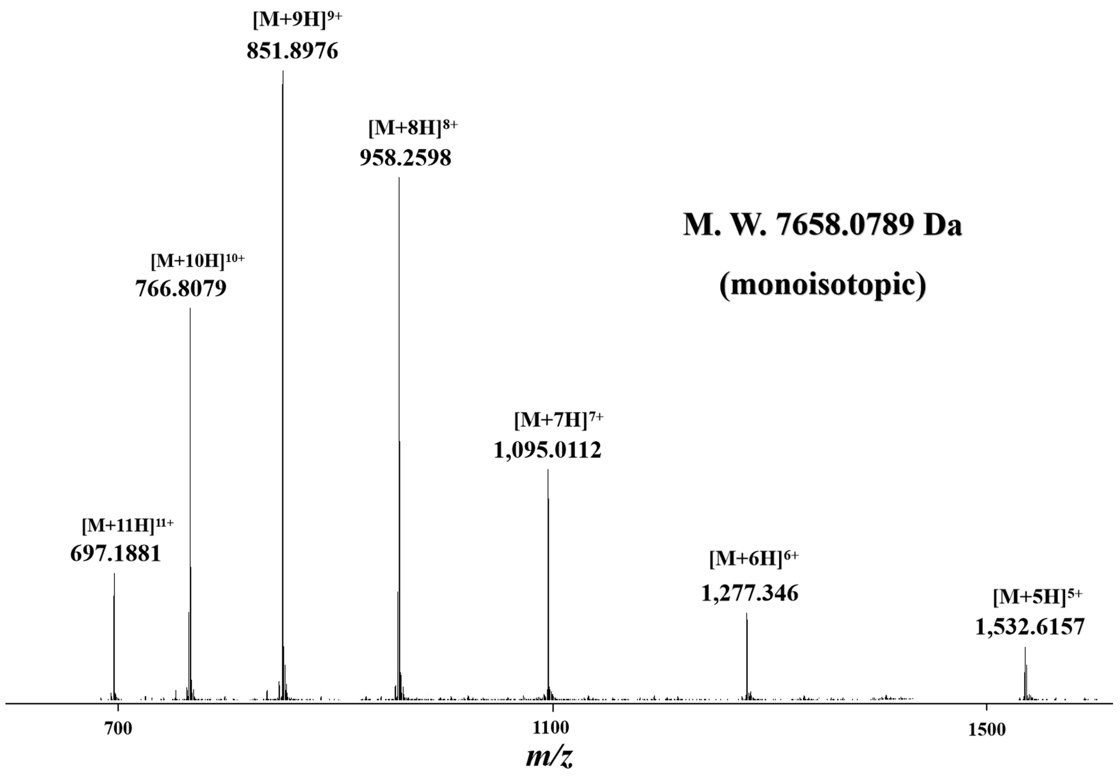

Figure S1. Q-TOF mass spectrum of cyanogen bromide treated toyoncin, showing the loss of formylmethionine. Multiple charged molecular ions detected are indicated. M.W., molecular weight.
